# Non-Invasive Risk Assessment and Prediction of Mortality in Patients Undergoing Coronary Artery Bypass Graft Surgery

**DOI:** 10.3390/jcdd10090365

**Published:** 2023-08-25

**Authors:** Ju-Youn Kim, Young-Jun Park, Kyoung-Min Park, Young-Keun On, June-Soo Kim, Seung-Jung Park, Young-Tak Lee

**Affiliations:** 1 Division of Cardiology, Department of Internal Medicine, Heart Vascular and Stroke Institute, Samsung Medical Center, Sungkyunkwan University School of Medicine, Seoul 06351, Republic of Korea; 2 Division of Cardiology, Department of Internal Medicine, Yonsei University Wonju College of Medicine, Wonju 24715, Republic of Korea; 3 Department of Thoracic and Cardiovascular Surgery, Incheon Sejong Hospital, Incheon 21080, Republic of Korea

**Keywords:** coronary artery bypass graft, heart rate turbulence, T-wave alternans, death, left ventricular systolic function

## Abstract

**Objectives:** Heart rate turbulence (HRT) and T-wave alternans (TWA), non-invasive markers of cardiac autonomic dysfunction, and ventricular repolarization abnormality, reportedly, predict the risk of cardiovascular death after myocardial infarction. We investigated whether pre-operative assessment of HRT and/or TWA could predict long-term mortality following coronary artery bypass graft (CABG) surgery. **Methods:** From May 2010 to December 2017, patients undergoing elective CABG and receiving 24 h ambulatory electrocardiogram monitoring 1 to 5 days prior to CABG surgery were prospectively enrolled. Pre-operative HRT and TWA were measured using a 24 h ambulatory electrocardiogram. The relative risk of cardiac or overall death was assessed according to abnormalities of HRT, TWA, or left ventricular ejection fraction (LV EF). **Results:** During the mean follow-up period of 4.6 ± 3.9 years, 40 adjudicated overall (5.9%/yr) and 5 cardiac deaths (0.9%/yr) occurred in 146 enrolled patients (64.9 ± 9.3 years; 108 males). Patients with abnormal HRT exhibited significantly higher relative risks of cardiac death (adjusted hazard ratio [HR] 24.9, 95% confidence interval [CI] 1.46–427) and all-cause death (adjusted HR 5.77, 95% CI 2.34–14.2) compared to those with normal HRT. Moreover, abnormal HRT plus abnormal TWA and LV EF < 50% was associated with a greater elevation in cardiac and overall mortality risk. The predictive role of abnormal HRT with/without abnormal TWA for all-cause death was likely more prominent in patients with mildly reduced (35 to 50%) or preserved (≥50%) LV EF. Abnormal HRT plus abnormal TWA and LV EF < 50% showed high negative predictive value in cardiac and overall mortality risk. **Conclusions:** Assessment of pre-operative HRT and/or TWA predicted mortality risk in patients undergoing elective CABG. Combined analysis of HRT, TWA, and LVEF enhanced the prognostic power. In particular, the predictive value of HRT was enhanced in patients with preserved or mid-range LV EF.

## 1. Introduction

Coronary artery bypass graft (CABG) surgery has been established as the treatment of choice for myocardial revascularization in patients with severe coronary artery disease (CAD) [1,2]. However, cardiovascular death remains the main cause of death in patients undergoing CABG, even after successful revascularization [3]. 

Therefore, more accurate risk stratification and better management of patients treated by CABG are critical in clinical practice. Left ventricular (LV) ejection fraction (EF) is the most widely used prognosis parameter after CABG surgery [4,5]. However, more than half of deaths after CABG occur in patients with preserved or moderately impaired LV systolic function, showing a low positive predictive value of LV EF [6,7]. Given the limitation of LV EF as a prognostic factor, continuous efforts are required to find additional risk stratifiers for patients undergoing CABG surgery.

Heart rate turbulence (HRT) and T-wave alternans (TWA), non-invasive electrocardiographic markers reflecting cardiac autonomic dysfunction and ventricular repolarization abnormality, respectively, have been shown to predict the risk of cardiovascular or SCD in patients with ischemic or non-ischemic cardiomyopathy [8,9,10,11,12,13]. Previously, we reported that pre-operative abnormal HRT was significantly associated with in-hospital new-onset atrial fibrillation (AF) and post-discharge AF recurrence or stroke after CABG surgery [14]. However, few prospective studies have described the prognostic value of HRT or TWA for predicting mortality in patients undergoing elective CABG surgery. 

Therefore, we investigated whether pre-operative abnormal HRT or TWA could predict post-CABG mortality using a prospective CABG registry. We also evaluated the prognostic value of the non-invasive parameters alone or in combination with LV EF. 

## 2. Materials and Methods

### 2.1. Study Population 

This was a prospective, single-center, observational registry study. We included consecutive patients who underwent elective CABG surgery with CAD, including acute or chronic coronary syndrome, and who received 24 h ambulatory electrocardiogram monitoring 1 to 5 days prior to CABG surgery from May 2010 to December 2017.

Inclusion criteria for the present study were as follows: (1) age ≥ 18 years; (2) CABG based on current guidelines; and (3) pre-CABG Holter recording for at least 20 h. Exclusion criteria for the present study were as follows: (1) urgent/emergent surgery prohibiting pre-operative 24 h electrocardiogram monitoring; (2) inadequate Holter recording for assessment of HRT or TWA; (3) pre-existing (permanent, persistent, or paroxysmal) AF; and (4) ventricular paced rhythm. This study was approved by the Institutional Review Board (IRB) of Samsung Medical Center in the Republic of Korea (IRB number: 2010-04-050). All patients provided informed consent.

### 2.2. Recording and Analysis of Ambulatory Electrocardiogram 

The recording and analysis of ambulatory electrograms have been described previously [14]. In brief, an ambulatory 24 h electrogram recording was performed using a 3-lead (V1, V3, and V5) SEER Light Digital Holter monitor with a sampling rate of 125 samples per second (GE Healthcare Inc., Milwaukee, WI, USA). HRT and TWA values were analyzed using a MARS 8000 Holter analyzer (GE Healthcare Inc., Milwaukee, WI, USA). HRT represents the biphasic change of heart rate following premature ventricular complex (PVC). Therefore, assessment of HRT is limited to patients with sinus rhythm with a sufficient number of PVCs at least 5 beats. HRT consists of initial acceleration and late deceleration of heart rate. Turbulence onset (TO) was defined as the percentage difference between the heart rate immediately following and immediately preceding a PVC. Turbulence slope (TS) was defined as the maximum positive regression slope for each sequence of five consecutive sinus rhythm R-R intervals within the first 15 intervals after a PVC [15]. Values of TO > 0% and TS < 2.5 ms/RR intervals were defined as abnormal. For the present study, HRT was defined as impaired when both TO and TS were abnormal. TWA was measured by a modified moving average analysis in 3 leads. The maximum value in 24 h was assessed with a cutoff of ≥47 µV defined as abnormal [12]. Manual editing was performed by an experienced physician, excluding rhythm strips with high noise levels, baseline undulation, or ventricular bigeminy. 

### 2.3. CABG Surgery

All surgeries were performed via standard median sternotomy. At our hospital, off-pump CABG via the bilateral internal thoracic arteries is the preferred technique. The internal thoracic artery was used as the main graft, and other vessels such as the great saphenous vein were used if needed. The type of surgery and treatment strategies including the number of grafts used and administration of medication after CABG were determined by the experienced surgeon. Aspirin or warfarin was started within the first 24 h after surgery and beta blockers, calcium channel blockers, renin–angiotensin system blocker, or anti-arrhythmic agents were used during the postoperative period when clinically indicated. 

## 3. Data Collection and Study Outcomes 

**Figure 1:**
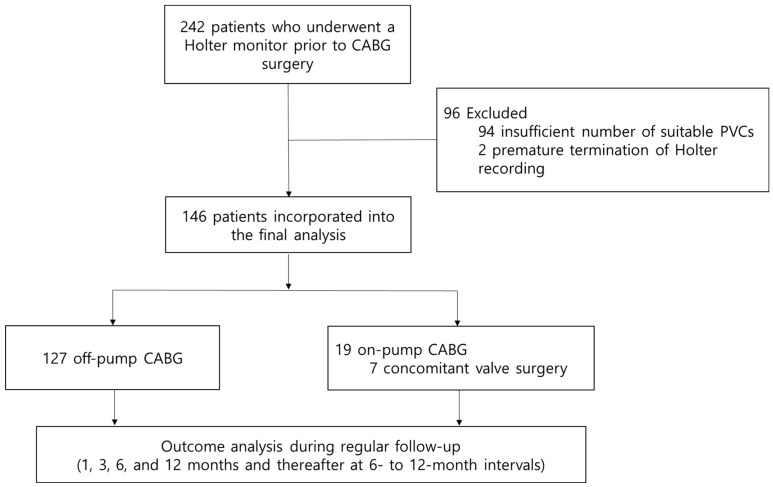
Flow chart of the study population. CABG: coronary artery bypass graft, PVC: premature ventricular complex.

We prospectively collected basic demographic data, past medical and medication history, 12-lead electrocardiographic and echocardiographic data, type or extent of coronary artery disease, and surgery-related data. The medication data was collected during the period of 24 h ambulatory electrocardiogram monitoring before CABG surgery. The primary outcome of this study was cardiac death including sudden cardiac death and all-cause death. Patients were regularly followed up in the outpatient clinic for at least 1 year (1, 3, 6, and 12 months and thereafter at 6- to 12-month intervals) or through telephone interviews when patients did not visit the clinic at the scheduled time (Figure 1). Masked adjudication of all relevant events was performed by expert physicians, who reviewed cases in a blinded manner regarding the study treatment. The relative risk of outcomes with abnormal HRT alone and in combination with abnormal HRT or LV systolic dysfunction (LV EF < 50%) was assessed. Subgroup analysis was performed according to the degree of baseline LV systolic function: (1) preserved EF (≥50%), (2) mid-range EF (35% ≤ EF < 50%), and 3) reduced EF (≤35%).

## 4. Statistical Analysis

Continuous variables are expressed as median with interquartile range, and categorical variables are presented as count and percentage and were compared by the Mann–Whitney test and Fisher’s exact test. Event rate curves were obtained using a Kaplan–Meier analysis and were compared using the log-rank test. The risks for the outcomes were assessed using a Cox proportional hazards model and are presented as adjusted hazard ratio (HR) with a 95% confidence interval (CI) for age, sex, medication use of beta blocker and renin–angiotensin system blocker, QRS duration, numbers of PVCs, and acute (vs. chronic) coronary syndrome. To assess the sensitivity and specificity of each parameter and to define the cut-off points that maximized the separation of outcome analysis, receiver operating characteristic analysis was performed. *p*-values < 0.05 were considered statistically significant. All statistical analyses were performed using the Statistical Package for the Social Sciences (SPSS) software, version 27.0 (SPSS, Inc., Chicago, IL, USA). 

## 5. Results

### 5.1. Clinical Characteristics

A total of 146 patients (mean age 64.9  ± 9.3 years; 108 [74.0%] males) were included in the present study. Of the 146 patients, 112 (76.7%) had three-vessel disease. Thirteen (8.9%) patients received CABG for acute coronary syndrome. The mean LV EF was 57.0 ± 12.6%. Fourteen (9.6%) patients showed LV EF less than 35% and 26 (17.8%) patients showed mid-range LV EF (35–50%). Off-pump CABG was performed in 125 (85.6%) patients. Among the patients who received on-pump CABG, 7 underwent concomitant valve surgery (five for mitral valvuloplasty and two for aortic valve replacement). Further, 57 (39.6%) patients were receiving beta blockers and 60 (41.1%) patients were receiving renin–angiotensin system blockers during the Holter exam. The mean TO was −0.6 ± 2.3 % (range, −10.6 to 10.3), the mean TS was 5.8 ± 7.1 ms/RR interval (range, −4.8 to 47.2), and the mean TWA was 46.2 ± 24.9 µV (range, 9.0–99.0). According to the predefined criteria, 15 (10.3%) patients were identified to have abnormal HRT and abnormal TWA was observed in 59 (40.4%) patients. The baseline characteristics of the patients are summarized in Table 1.

### 5.2. Clinical Events

**Figure 2:**
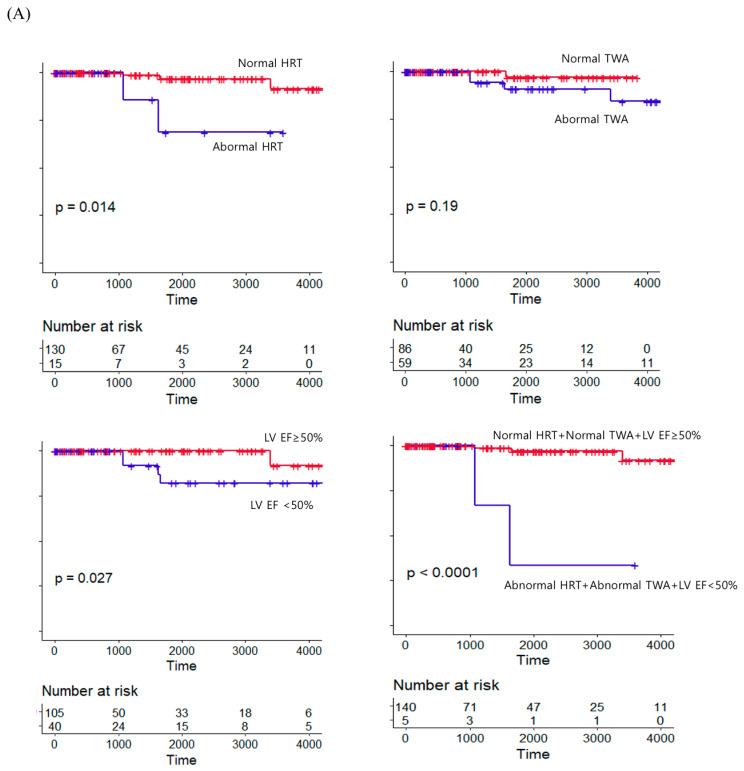

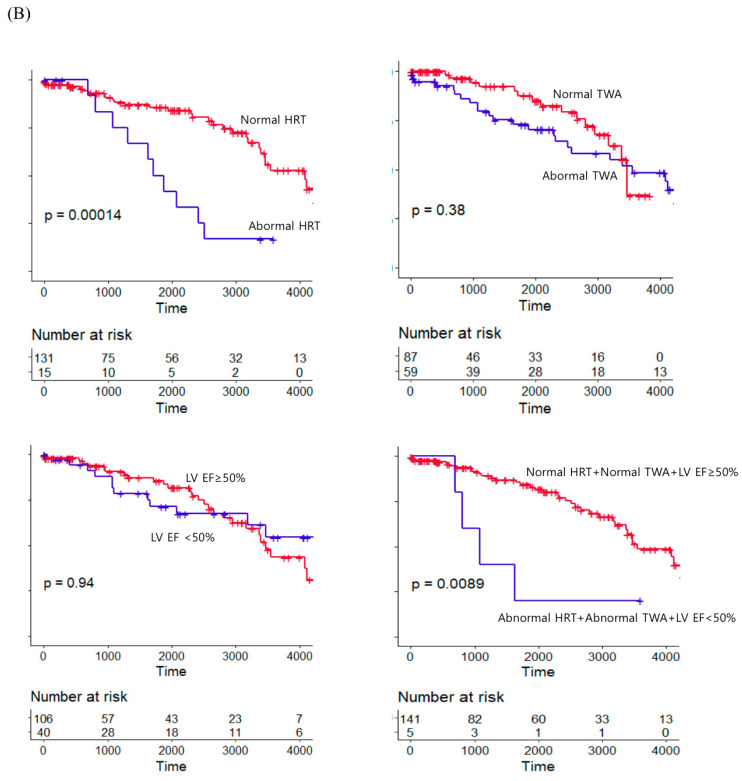
Survival curves of cardiac death (**A**) and all-cause death (**B**) according to each or various combinations of variables including heart rate turbulence, T-wave alternans, and impaired ejection fraction. HRT: heart rate turbulence, TWA: T-wave alternans, LV EF: left ventricular ejection fraction.

Forty adjudicated overall deaths (event rate 5.9%/yr) and 5 cardiac deaths (event rate 0.9%/yr) occurred during the mean follow-up duration of 4.6 ± 3.9 years. A multivariate analysis was performed adjusted for age, sex, medication use of beta blocker and renin–angiotensin system blocker, QRS duration, numbers of PVCs, and acute (vs. chronic) coronary syndrome. The abnormal HRT group showed a significantly higher relative risk of cardiac death (adjusted HR 24.9, 95% CI 1.46–427) and all-cause death (adjusted HR 5.77, CI 2.34–14.2) compared to the normal HRT group (Table 2). In contrast, abnormal TWA or LV systolic dysfunction (<50%) alone showed no clinical significance for predicting cardiac or all-cause death. When LV EF was evaluated as a continuous variable, higher LV EF was likely to show a reduced risk for cardiac death in univariate analysis (HR 0.94, 95% CI 0.89–0.99), but its clinical significance did not persist in multivariate analysis (adjusted HR 0.66, 95% CI 0.15–2.85). However, patients with triple abnormalities, including abnormal HRT, abnormal TWA, and LV EF < 50%, were associated with greater risk elevation; there was an 87.2-fold (95% CI 1.97–3860) increase in cardiac death and a 9.20-fold (95% CI 2.73–31.0) increase in overall mortality risk (Table 2). A worse prognosis was also shown by survival curves constructed depending on abnormal values of HRT, TWA, and LV EF (Figure 2). Additional analysis was performed except for those who underwent concomitant valve surgery. Overall, consistent results were obtained when analyzed with the patients who underwent lone CABG surgery separately. The abnormal HRT group showed a significantly higher risk of all-cause death (adjusted HR 7.39, 95% CI 2.88–19.00) compared to the normal HRT group. Abnormal TWA or LV EF < 50% showed no clinical significance for predicting all-cause death. Abnormal HRT and abnormal TWA and LV EF < 50% group were associated with a significantly higher risk of all-cause death (adjusted HR 9.70, 95% CI 2.86–32.68) (Table 3). 

The accuracy values were higher for a combination of abnormal HRT, abnormal TWA, and LV EF < 50% (0.96 for cardiac death, 0.75 for all-cause death). All parameters showed high specificity and negative predictive values (Table 4).

**Figure 3:**
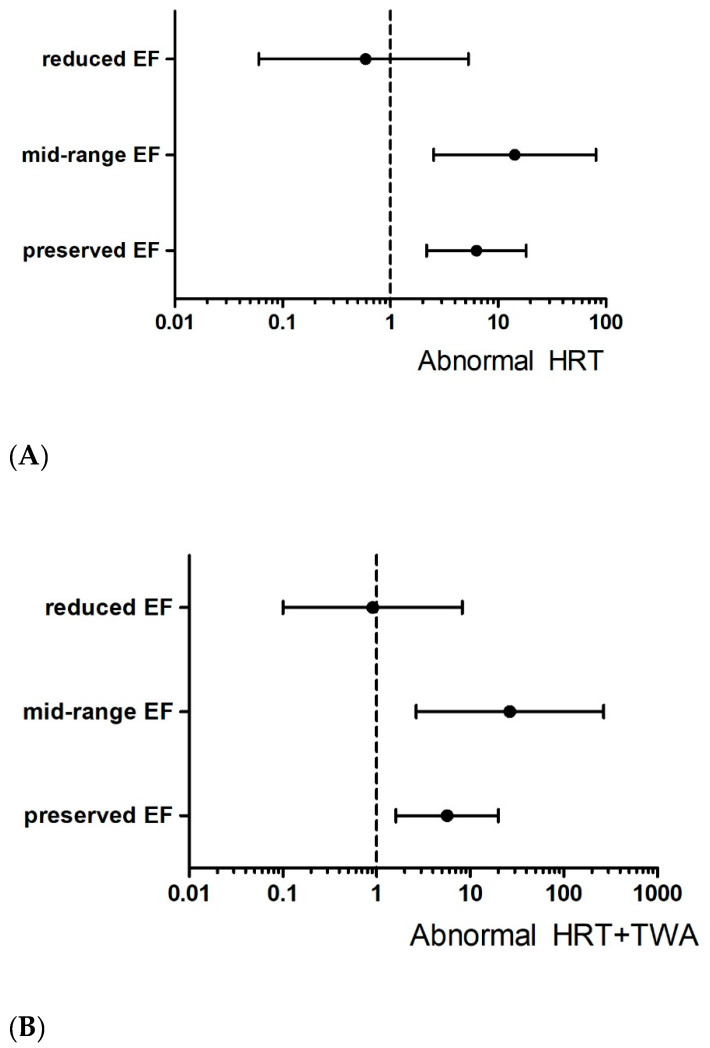
Hazard ratio of all-cause death according to left ventricular systolic function: (**A**) abnormal heart rate turbulence and (**B**) both abnormal heart rate turbulence and T-wave alternans. EF: ejection fraction, HRT: heart rate turbulence, TWA: T-wave alternans.

The subgroup analysis was performed according to baseline LV EF (severely reduced, mid-range, and preserved EF). Incidence rates of all-cause death were 6.53%, 5.91%, and 5.79% per year in the severely reduced, mid-range, and preserved EF groups, respectively. Abnormal HRT with/without abnormal TWA played no significant role in predicting all-cause death in the severely reduced EF group (HR 0.59, 95% CI 0.06–5.30). However, the predictive value of abnormal HRT with/without abnormal TWA regarding all-cause mortality became evident in the mid-range (HR 14.3, 95% CI 2.52–81.0 for abnormal HRT) and preserved EF groups (HR 6.3, 95% CI 2.17–18.3 for abnormal HRT) (Figure 3).

## 6. Discussion

### 6.1. Main Findings

This study investigated the predictive value of pre-operative ambulatory ECG-based non-invasive parameters for serious outcomes after elective CABG surgery in CAD patients during a mean follow-up period longer than 4 years. Abnormal HRT measured within 5 days prior to surgery predicted hard outcomes during long-term follow-up. The combination of abnormal HRT, abnormal TWA, and impaired EF improved the predictive value up to 9 fold for all-cause death and 87 fold for cardiac death. This predictive value was particularly significant in patients with preserved or mid-range LV systolic function. Furthermore, the negative predictive value of HRT was high enough to predict cardiac death. The accuracy goes higher when analyzing combined parameters including abnormal HRT, abnormal TWA, and abnormal LV function compared to single each parameter. 

### 6.2. Prognostic Value of TWA and HRT

Severe LV systolic dysfunction is the most powerful prognostic factor for cardiovascular or SCD in patients with ischemic or non-ischemic cardiomyopathy, for whom an implantable cardioverter defibrillator (ICD) is recommended by the current guidelines to reduce the risk of mortality [16]. However, almost half of the deaths have reportedly occurred in post-MI patients with preserved or mildly reduced LV EF [7,17]. Therefore, identifying additional predictors associated with a worse outcome is particularly important in this kind of patient population with stable ischemic heart disease.

HRT, a surrogate marker of cardiac autonomic dysfunction, has been proposed as a useful non-invasive prognostic marker for mortality in patients with MI [11,18]. HRT represents a short-term fluctuation in sinus cycle length that follows PVC. To overcome masking by heart rate variability, averaging responses to the number of PVCs is needed to characterize the pattern, and HRT values were corrected for heart rate or a certain number of PVCs. Indeed, in our data, patients with pre-CABG abnormal HRT values exhibited a greater cardiac and overall mortality rate than those with normal HRT. The prognostic significance of pre-operative HRT has already been demonstrated by Cygankiewicz et al. in 111 patients undergoing CABG; patients with abnormal HRT had a 3.36-fold higher risk of cardiac death than those with normal HRT [19]. However, they followed up with patients only for 1 year and it was not clear how many patients underwent off-pump or on-pump CABG. Moreover, they did not evaluate the prognostic value of HRT in combination with LV systolic function or in subgroups divided by LV systolic function. In contrast, we follow up patients for a much longer period of time (4.6 ± 3.9 years), during which the predictive power of HRT was well maintained. The prognostic significance of the HRT was augmented when assessed combined with LV EF and/or TWA. 

### 6.3. Clinical Implications

Previously, clinical implications of HRT and/or TWA were validated mostly in patients with reduced LV systolic function [8,9,10,11,12,13,14,15]. Additionally, several trials have suggested HRT and/or TWA may be predictors for SCD in patients with mildly reduced LV systolic function after MI [11,20,21]. However, patients with preserved LV EF (EF ≥ 50%) were not included in the studies. HRT is known as influenced by LV EF. HRT indexes are significantly decreased in patients with heart failure including preserved LV systolic function compared to the normal population. Interestingly, our study demonstrated that the predictive role of HRT was more evident in patients with mildly reduced or preserved EF, for whom no specific indications have been proposed for prophylactic ICD therapy, although the absolute number of SCDs is much higher in this population than in those with reduced LV EF. Recently, patients undergoing coronary revascularization have tended to have better LV systolic function due to more advanced diagnostic tests, surgical skills, and management strategies. Indeed, the average LV EF of our patients was 57 ± 13% and more than 85% of them underwent off-pump CABG which has lesser postoperative complications than on-pump CABG. Therefore, our findings may have more meaningful clinical implications for better risk stratification of patients with mildly reduced or preserved EF. Furthermore, our study cohort included ischemic heart disease who had complex coronary lesions and consisted of less than 10% of the ACS population. In other words, our study cohort itself had low mortality risk or sudden cardiac death risk compared to other studies with the MI population. Nevertheless, impaired HRT and TWA showed high predictive value in identifying patients at increased risk of death. Therefore, careful follow-up and management should be necessary for more than 5 years in this population. 

In our study result, the negative predictive value of combined parameters including abnormal HRT plus abnormal TWA plus LV EF < 50% was high, especially in cardiac death (98%). The positive predictive value of combined parameters showed low in both outcomes. The accuracy was higher when analyzing with combined parameters than a single parameter alone. These results are similar to previous studies [11,20]. The combination of these parameters can be used to assess risk stratification in high specificity.

Recently, the utilization of single- or multi-channel wearable devices for ambulatory electrocardiographic monitoring has gained unprecedented interest from physicians and patients [22,23]. Various algorithms to assess HRT and/or TWA can be incorporated into such wearable devices, making it more feasible to assess the clinical value of non-invasive variables in a greater number of patients with a relatively preserved LV systolic function. 

The optimal time for HRT measurement is not definitive; serial testing has shown that non-invasive tests beyond 8 weeks after MI reliably identify patients at risk of serious events. The REFINE study reported that the combination of impaired HRT and TWA measured at 10 to 14 weeks post-MI can predict hard outcomes in patients with impaired LV systolic function [20]. However, in our study, TWA and HRT values measured before elective surgery were significantly associated with cardiac or overall death, suggesting that not only post-operative but also pre-operative assessment of baseline cardiac autonomic dysfunction or repolarization abnormality are of clinical value in predicting hard outcomes. In a previous study, HRT and TWA testing at 2 to 4 weeks after MI did not reliably identify patients at risk [20]. This might be due to a change in autonomic tone immediately after MI decreased the prognostic power. Our result suggests that the baseline autonomic tone is more effective in predicting the hard outcomes.

### 6.4. Limitations

This study was a prospective observational study, but the sample size and number of events, particularly cardiac deaths, were small. Assessment of HRT is limited to patients with sinus rhythm and VPC with certain VPC characteristics and R-R patterns. Therefore, some patients were excluded from the final analysis. The lower cardiac event rate may be related to complete revascularization therapy with CABG of most patients with preserved baseline LV systolic function. To overcome this limitation, we observed patients for a long period of time and analyzed all-cause mortality as well. Also, this study missed other risk stratification algorithms such as the European System for Cardiac Operative Risk Evaluation (EuroSCORE), which is a cardiac risk model for predicting mortality after cardiac surgery. In addition, the risk of SCD was not assessed due to the low event rate. Therefore, additional studies with a larger scale should be conducted to validate the clinical implications of HRT and/or TWA for risk stratification of arrhythmic or SCD in patients undergoing CABG surgery.

## 7. Conclusions 

Pre-operative measurement of HRT and/or TWA was likely useful in risk stratification of mortality in patients undergoing elective CABG. In particular, the predictive value was significant in patients with preserved or mid-range reduced LV EF. Additionally, the combined analysis of HRT, TWA, and LV EF improved the prognostic power. These parameters might be used for screening the high-risk population for cardiac adverse outcomes. Further, a larger-scale trial is necessary to confirm the clinical implications.

## Figures and Tables

**Table 1:** Clinical characteristics of the study population.

	Total (*n* = 146)	Cardiac Death (*n* = 5)	All-Cause Death (*n* = 40)	Alive (*n* = 106)
Age, years	64.9 ± 9.3	68.8 ± 5.2	69.5 ± 6.3	63.2 ± 9.6
Male, *n* (%)	108 (74.0%)	4 (80.0%)	32 (80%)	76 (71.7%)
Hypertension, *n* (%)	105 (71.9%)	5 (100%)	35 (87.5%)	70 (66.0%)
Diabetes, *n* (%)	73 (50.0%)	4 (80.0%)	19 (47.5%)	54 (50.9%)
PCI, *n* (%)	22 (15.1%)	0	5 (12.5%)	17 (16.0%)
Heart failure, *n* (%)	8 (5.5%)	2 (40.0%)	3 (7.5%)	5 (4.7%)
CKD, *n* (%)	5 (3.4%)	0	3 (7.5%)	2 (1.9%)
Stroke, *n* (%)	25 (17.1%)	2 (40.0%)	12 (30.0%)	13 (12.3%)
OP CABG, *n* (%)	125 (85.6%)	2 (40.0%)	33 (82.5%)	92 (86.8%)
Index diagnosis				
ACS, *n* (%)	13 (8.9%)	2 (40.0%)	4 (10.0%)	9 (8.5%)
CCS, *n* (%)	133 (91.1%)	3 (60.0%)	36 (90.0%)	97 (91.5%)
RAS blocker, *n* (%)	60 (41.1%)	4 (80.0%)	19 (47.5%)	41 (38.7%)
Beta blocker, *n* (%)	57 (39.6%)	2 (40.0%)	17 (42.5%)	40 (38.5%)
LV EF, %	57.0 ± 12.6	39.5 ± 12.4	54.9 ± 12.2	57.8 ± 12.8
TO, %	−0.6 ± 2.3	0.7 ± 1.4	−0.1 ± 2.6	−0.8 ± 2.1
TS, ms/RR interval	5.8 ± 7.1	1.8 ± 1.8	4.5 ± 6.7	6.3 ± 7.2
TWA, µV	46.2 ± 24.9	69.6 ± 33.8	56.7 ± 28.0	42.2 ± 22.5

PCI: percutaneous coronary intervention, CKD: chronic kidney disease, OP CABG: off-pump coronary artery bypass graft, ACS: acute coronary syndrome, CCS: chronic coronary syndrome, RAS: renin–angiotensin system, LV EF: left ventricular ejection fraction, TO: turbulence onset, TS: turbulence slope, TWA: T-wave alternans.

**Table 2:** Hazard ratios of outcomes by variables.

Subjects (N = 146)	Cardiac Death (N = 5, Event Rate 0.9%/yr)		All-Cause Death (N = 40, Event Rate 5.9%/yr)	
	HR (95% CI)	HR * (95% CI)	HR (95% CI)	HR * (95% CI)
Abnormal HRT	6.89 (1.15–41.4)	24.9 (1.46–427)	3.77 (1.81–7.85)	5.77 (2.34–14.2)
Abnormal TWA	3.99 (0.44–36.4)	7.47 (0.47–119)	1.35 (0.69–2.62)	1.39 (0.68–2.85)
LV EF < 50%	8.06 (0.90–72.3)	NA	0.98 (0.50–1.90)	1.29 (0.61–2.73)
LV EF (continuous)	0.94 (0.89–0.99)	0.66 (0.15–2.85)	1.00 (0.98–1.02)	0.98 (0.95–1.01)
Abnormal HRT + Abnormal TWA	10.3 (1.72–62.2)	27.3 (1.68–442)	4.31 (1.88–9.89)	5.14 (1.92–13.8)
Abnormal HRT + Abnormal TWA + LV EF < 50%	18.3 (2.97–113)	87.2 (1.97–3860)	3.67 (1.29–10.4)	9.2 (2.73–31.0)

HR: hazard ratio, CI: confidence interval, HRT: heart rate turbulence, TWA: T-wave alternans, LV EF: left ventricular ejection fraction, NA: not applicable. * adjusted for age, sex, medication use of beta blocker and renin–angiotensin system blocker, QRS duration, numbers of premature ventricular complexes, and diagnosis (acute vs. chronic coronary syndrome).

**Table 3:** Hazard ratios of outcomes by variables except who underwent valve surgery.

Subjects (N = 139)	Cardiac Death (N = 4, Event Rate 0.7%/yr)		All-Cause Death (N = 39, Event Rate 6.2%/yr)	
	HR (95% CI)	HR * (95% CI)	HR (95% CI)	HR * (95% CI)
Abnormal HRT	13.71 (1.91–98.29)	NA	4.63 (2.20–9.77)	7.39 (2.88–19.00)
Abnormal TWA	2.69 (0.27–27.00)	NA	1.23 (0.63–2.40)	1.27 (0.62–2.59)
LV EF < 50%	6.89 (0.71–66.45)	NA	1.01 (0.51–2.00)	1.26 (0.59–2.71)
LV EF (continuous)	0.94 (0.88–0.99)	0.55 (0.10–3.13)	0.99 (0.97–1.02)	0.97 (0.94–1.01)
Abnormal HRT + Abnormal TWA	14.4 (2.02–102.70)	NA	4.16 (1.81–9.57)	5.87 (2.08–16.54)
Abnormal HRT + Abnormal TWA + LV EF < 50%	26.20 (3.52–194.86)	NA	3.50 (1.23–9.97)	9.70 (2.86–32.68)

HR: hazard ratio, CI: confidence interval, HRT: heart rate turbulence, TWA: T-wave alternans, LV EF: left ventricular ejection fraction, NA: not applicable. * adjusted for age, sex, medication use of beta blocker and renin–angiotensin system blocker, QRS duration, numbers of premature ventricular complexes, and diagnosis (acute vs. chronic coronary syndrome).

**Table 4:** Sensitivity, specificity, and predictive accuracy of the parameters to predict the outcomes (cardiac death/all-cause death).

Parameters		Sensitivity	Specificity	PPV	NPV	Accuracy
Abnormal HRT	Cardiac death	0.40	0.91	0.13	0.98	0.89
	All-cause death	0.25	0.95	0.67	0.77	0.76
Abnormal HRT + abnormal TWA	Cardiac death	0.40	0.96	0.25	0.98	0.94
	All-cause death	0.18	0.99	0.88	0.76	0.77
Abnormal HRT + abnormal TWA + LV EF < 50%	Cardiac death	0.40	0.98	0.40	0.98	0.96
	All-cause death	0.10	0.99	0.80	0.74	0.75

PPV: positive predictive value, NPV: negative predictive value, HRT: heart rate turbulence, TWA: T-wave alternans, LV EF: left ventricular ejection fraction.

## Data Availability

The data that support the findings of this study are available from the corresponding author upon reasonable request.
